# Leaf stomatal traits rather than anatomical traits regulate gross primary productivity of moso bamboo (*Phyllostachys edulis*) stands

**DOI:** 10.3389/fpls.2023.1117564

**Published:** 2023-03-14

**Authors:** Wen Guo, Paolo Cherubini, Jian Zhang, Mai-He Li, Lianghua Qi

**Affiliations:** ^1^ Key Laboratory of National Forestry and Grassland Administration/Beijing Bamboo & Rattan Science and Technology, International Centre for Bamboo and Rattan, Beijing, China; ^2^ Forest Dynamics, Swiss Federal Institute for Forest, Snow and Landscape Research WSL, Birmensdorf, Switzerland; ^3^ Department of Forest and Conservation Sciences, Faculty of Forestry, University of British Columbia, Vancouver, BC, Canada; ^4^ Key Laboratory of Geographical Processes and Ecological Security in Changbai Mountains, Ministry of Education, School of Geographical Sciences, Northeast Normal University, Changchun, China; ^5^ School of Life Science, Hebei University, Baoding, China

**Keywords:** bamboo productivity, environment effects, leaf anatomy, leaf traits, structural equation modeling, trait coordination network

## Abstract

Leaf stomatal and anatomical traits strongly influence plant productivity. Understanding the environmental adaptation mechanisms of leaf stomatal and anatomical traits and their relationship with ecosystem productivity is essential to better understand and predict the long-term adaptation strategies to climate change of moso bamboo forests. Here, we selected 6 sites within the moso bamboo distribution area, measured 3 leaf stomatal traits and 10 leaf anatomical traits of unmanaged moso bamboo stands. We explored the spatial variation characteristics of these traits and their response to environmental changes, assessed the relationships among these traits at regional scales through network analysis, and tested the direct and indirect effects of environmental, leaf stomatal and anatomical traits on gross primary productivity (GPP) of bamboo stands using structural equation modeling (SEM). The results showed that both climate and soil factors significantly affected leaf stomatal and anatomical traits of moso bamboo. Solar radiation (SR) and mean annual precipitation (MAP) out of the climatic factors were the key drivers of variation in leaf stomatal and anatomical traits, respectively. Soil moisture and nutrients out of the soil properties significantly affected both leaf stomatal and anatomical traits of moso bamboo. Network analysis further indicated that there was a significant correlation between leaf stomata and anatomical traits. Stomatal size (SS) showed the highest centrality value at the regional scale, indicating that it plays a key role in adjusting the adaptation of plants to external environmental conditions. SEM analysis showed that environment did not directly but indirectly affect GPP *via* stomatal performance. The environment explained 53.3% and 39.2% of the variation in leaf stomatal and anatomical traits, respectively, and leaf stomatal traits explained 20.8% of the regional variation in GPP. Our results demonstrate a direct effect of leaf stomatal traits rather than leaf anatomical traits on bamboo ecosystem productivity, which provides new insights into model predictions of bamboo forests under global climate change.

## 1 Introduction

Stomata determine photosynthetic performance and transpiration efficiency by regulating the exchange of carbon and gases between plants and the external environment (Katul et al., 2010; Lavergne et al., 2020). Stomatal response to the environment can be divided into short-term (control of the stomatal opening and closing) and long-term adaptation (changes in morphological traits of stomata) (Casson and Gray, 2008; Haworth et al., 2013). Environmental factors such as light intensity, altitude, CO_2_ concentration, and water and nutrient availability may lead to changes in stomatal traits (Poole et al., 2000; Zhou et al., 2012; Driesen et al., 2020). For example, the stomatal density (SD) of *Betula papyrifera* Marsh. in Canada was positively correlated with mean annual precipitation (MAP) (Pyakurel and Wang, 2014). A study showed that SD and stomatal size (SS) of *Quercus suber* populations in East Asia were negatively correlated with MAP, while SS was negatively correlated with mean annual temperature (MAT) (Du et al., 2021). Sun et al. (2021) found that leaf stomatal traits were significantly positively correlated with soil properties such as soil nutrients (C, N content). Moreover, stomatal traits determine the growth ability of plants in a specific environment, which in turn affects ecosystem function (Reichstein et al., 2014; Wang et al., 2015). For instance, leaf stomatal traits of 394 species from tropical to cold temperate zones showed that stomatal traits significantly affected the variation in gross primary productivity (GPP) (Liu et al., 2020). However, the mechanisms of spatial variation in stomatal traits of monocotyledons and their relationship with ecosystem productivity are not clear.Plants can respond to external environmental changes by adjusting leaf anatomical traits during their development, which are essential for their own survival and competition with other plants (Sultan, 2000; Oguchi et al., 2006; Aguraijuja et al., 2015; Oguchi et al., 2018). An analysis covering 916 plant species from nine sample sites in China showed that MAT and MAP were the main factors controlling spatial variation of leaf anatomical traits (He et al., 2017). Lin et al. (2021) found that leaf anatomical traits of *Cyclobalanopsis* species were significantly negatively correlated with soil pH value. Moreover, leaf anatomical traits determine light absorption, gas exchange, and water transport, and plants can optimize their productivity by adjusting leaf anatomical traits (Adams et al., 2018). It was shown that plants could improve the internal light scattering and carbon assimilation capacity of leaves by increasing the thickness of spongy tissues; GPP was positively correlated with spongy tissue–leaf thickness ratio (ST/LT) and negatively correlated with palisade–spongy tissue ratio (PT/ST) at the community level (Terashima et al., 2011; He et al., 2017). It should be noted that monocotyledons may adapt to the external environment through their own specific anatomical structures. For example, monocotyledons can reduce water loss and increase the reflection of strong light by adjusting anatomical traits such as mesophyll, cuticle and epidermis thickness, and increase the diameter of vessels and vascular bundles to improve water transport capacity (Kou et al., 2019).

Investigating the coordinated relationships between stomatal traits (at the cellular level) and anatomical traits (at the tissue level) can help to understand the adaptation strategies of plants to heterogeneous environments, and predict the response of plants to climate change (Osunkoya et al., 2014; Stanisci et al., 2020; Sellin et al., 2022). Traits do not exist alone, they form multiple trait combinations to achieve their functions and enable plants to develop ecological strategies (Donovan et al., 2011; Bruelheide et al., 2018; Damiano et al., 2022). For instance, there were significant correlations between leaf anatomy (fenestrations and spongy tissue thickness) and stomatal traits (stomatal density and stomatal length) at different canopy positions of poplar clones (Li et al., 2015). On the contrary, Sellin et al. (2022) in *Populus tremula* and *P. tremuloide* found that there was no significant correlation between leaf stomata and anatomical traits. However, these studies have focused on whole forest communities or dicotyledons, and how stomatal and anatomical traits of monocotyledons are coordinated to adapt to different environments is still unexplored.

Moso bamboo is the most widely distributed and economically valuable bamboo species in China. Bamboos are clonal plants with fast horizontal clonal growth and expansion ability (Isagi et al., 2016; Song et al., 2016). According to the Ninth National Forest Resources inventory (2014-2018), the areas designated for moso bamboo in China have reached 4.68 million hm^2^, which accounts for 72.96% of the total bamboo forest area in China. Previous studies of leaf stomatal and anatomical traits of moso bamboo mainly focused on taxonomy and adaptation to stressful environments, such as cold and drought, yet little research has been conducted on unmanaged moso bamboo at large space-scales (Leandro et al., 2016; Wang et al., 2022).

In this study, we set up 24 plots in the main distribution areas of moso bamboo, focusing on the relationship between leaf stomatal and anatomical traits and abiotic environmental factors, and established the link between leaf traits (stomatal and anatomical) and ecosystem productivity. We hypothesize that 1) leaf stomatal traits rather than leaf anatomical traits play a more important role in determining the coordination network of traits in relation to environment changes, due to the functions of stomata in regulating plant water and carbon balance, and 2) environment factors (climatic and soil conditions) directly determine leaf stomatal and anatomical traits of moso bamboo, which further affect the variation of GPP at regional scale.

## 2 Materials and methods

### 2.1 Study site

**Figure 1:**
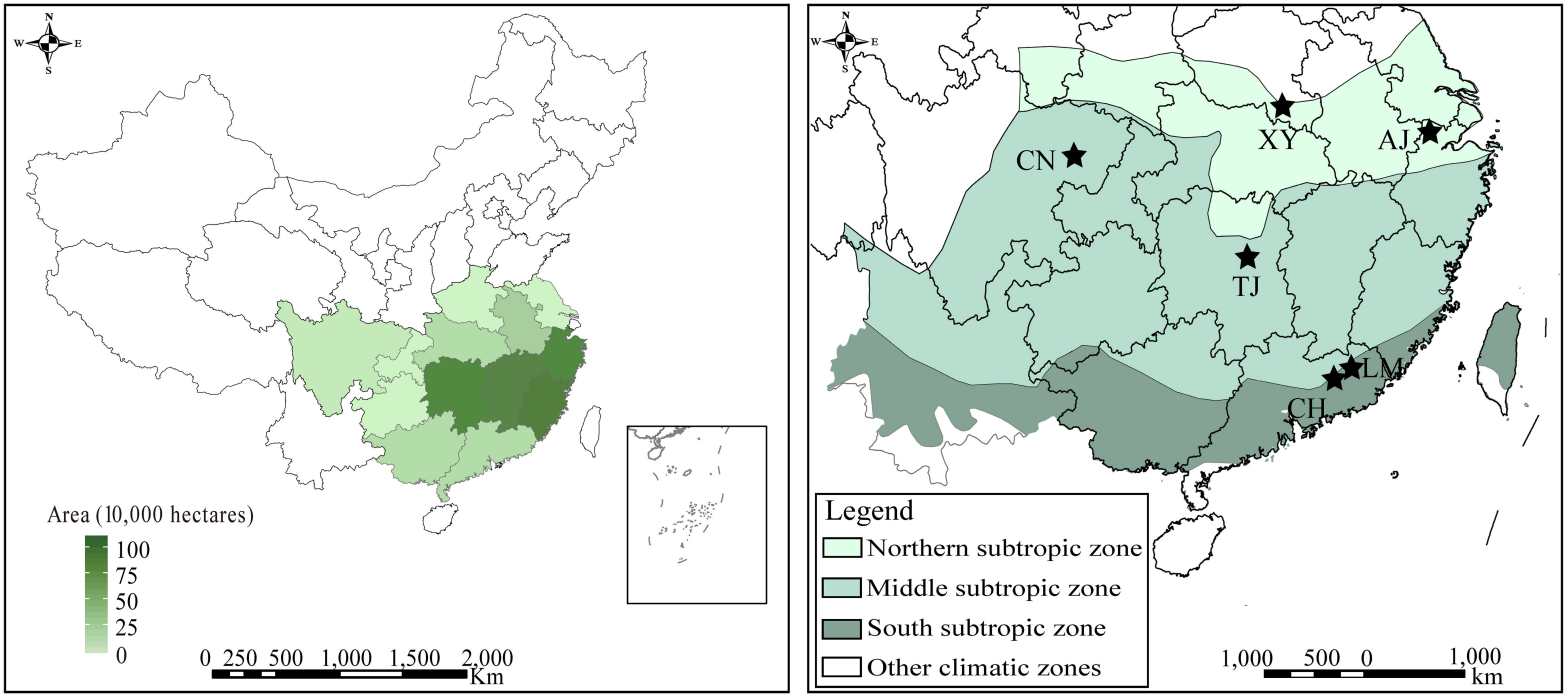
Distribution area and sampling site locations of moso bamboo.

The field experiment was conducted in the subtropical zones of China, which represent the main distribution areas of moso bamboo forests: Northern subtropic zone; including Xinyang (XY), Anji (AJ); Middle subtropic zone; including Changning (CN), Taojiang (TJ); South subtropic zone; including Conghua (CH), Longmen (LM) (**Figure 1**). Those moso bamboo forests range in latitude from 23°N to 31°N, and in longitude from 105°E to 119°E. The plots ranged from 14.2 to 20.2°C in mean annual temperature (MAT) and from 910 to 1510 mm in mean annual precipitation (MAP). The diameter at breast height of moso bamboo ranged from 7.1 to 10.7 cm, and the tree height ranged from 9.0 to 14.4 m. Those moso bamboo forests range in GPP from 0.98 to 1.69 kg C m^-2^ yr^-1^, detailed site information of the study areas is shown in **Table S1**.

### 2.2 Experimental design

In July of 2019, a total of 24 plots (6 site × 4 plots) were established in subtropical zone. To minimize human interference, the sampling plots were set up in unmanaged, unlogged areas, representing a typical bamboo forest ecosystem. Each sample plot was 20 m × 20 m, and the distance between sample plots was more than 1000 m to avoid spatial pseudoreplication. In total of 120 (6 site × 4 plots × 5 replications) new culms were selected.

### 2.3 Measurement of stomatal and anatomical traits

**Figure 2:**
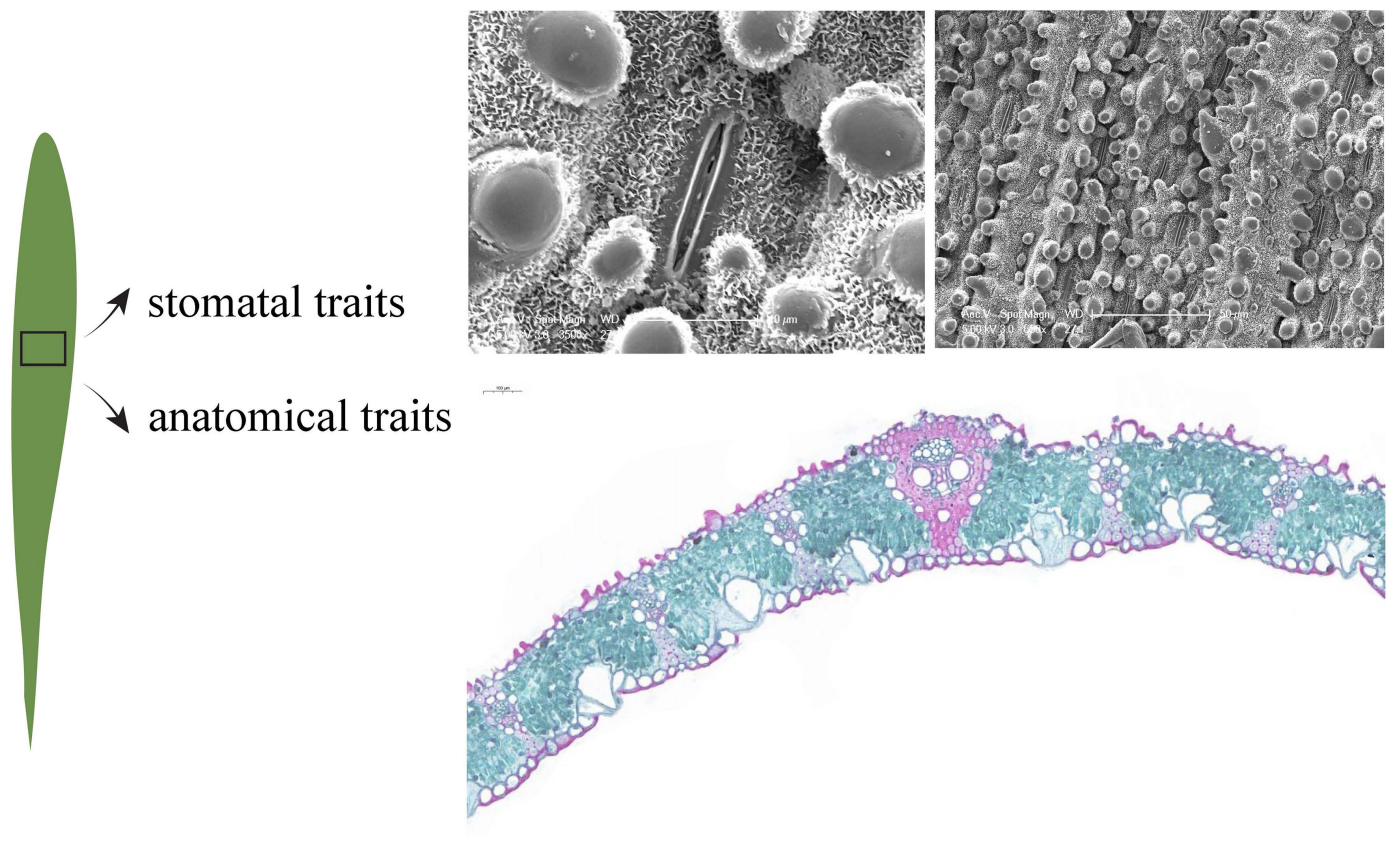
Images of leaf stomata and anatomy.


Twenty mature and healthy leaves were collected from the middle of the canopy on the south-facing side of each bamboo, and small pieces (1.0 × 0.5 cm) containing the midrib and part of the leaf were immediately cut from the leaves and fixed in FAA fixative (90 ml 75% ethanol + 5 ml formalin + 5 ml glacial acetic acid + 5 ml glycerol) for storage. The stomatal traits of leaves were observed using a scanning electron microscope (XL30-ESEM, Philips, Holland). Three small pieces were randomly selected from the fixed samples, and three photos were randomly taken at 650× and 3500× magnification, respectively, and the number of stomata in each photo was recorded (N_Photo_). Stomatal length (SL, µm) and stomatal width (SW, µm) were measured using Image J software (National Institutes of Health, Bethesda, MD, U.S.A.), and a total of 2160 photos were taken, and about 1080 stomata measured. The stomatal density (SD), stomatal size (SS) and stomatal relative area (SRA) of the leaves were calculated as follows (**Table 1**, **Figure 2**):

**Table 1:** Leaf stomatal and anatomical traits and their ecological strategies.

Traits	Abbreviation	Unit	Ecological strategy	Group
Stomatal density	SD	mm^2^	Gas exchange	Stomata
Stomatal size	SS	µm^2^	Gas exchange	Stomata
Stomatal relative area	SRA	%	Gas exchange	Stomata
Sectional area of first-vascular bundle	SAFVB	µm^2^	Transport, structure, defense	Anatomy
Sectional area of phloem	SAP	µm^2^	Transport, structure, defense	Anatomy
Sectional area of xylem	SAX	µm^2^	Transport, structure, defense	Anatomy
Sectional area of second-order vascular bundle	SASVB	µm^2^	Transport, structure, defense	Anatomy
Distance between adjacent vascular bundle	DBAVB	μm	Transport, structure, defense	Anatomy
Upper epidermal thickness	UET	μm	Transport, structure, defense	Anatomy
Lower epidermal thickness	LET	μm	Gas exchange, defense	Anatomy
Stratum corneum thickness	SCT	μm	Structure, defense	Anatomy
Mastoid process thickness	MPT	μm	Structure, defense	Anatomy
Sectional area of bulliform cell	SABC	µm^2^	Structure, defense	Anatomy

$$\text{SD}={\text{N}}_{\text{photo}}/\text{image area}$$

SS=π×SL×SW/4

$$\text{SRA}=\text{SS}\times \text{SD}\times 10^{-4}$$

Leaf samples were gradually dehydrated through a series of ethanol (50-100%) and permeated with paraffin. Leaf samples of approximately 10 μm in size were obtained with a slicer (Leica, RM2016, Germany) and then stained with Safranin O-Fast Green. Then, the anatomical structures were observed and photographed under a light microscope (ECLIPSE E100, NIKON, Japan) and the following 10 leaf anatomical traits were calculated using CaseViewer software 2.3 (3DHistech, Budapest, Hungary) (**Table 1**, **Figure 2**).

### 2.4 Soil sampling and analyses

After removal of the understory litter, soil samples were collected using a soil auger. A total of 72 soil samples (6 site × 4 plots × 3 replications) were collected from 0-20 cm soil layer. Each replicate was a soil mixture collected in each plot using a five-point sampling method. All soil samples were mixed and sieved through 100 mesh to remove large components, such as visible roots, debris, leaves. We analyzed soil bulk density (SBD), water content (SWC), pH, and concentrations of total carbon (TC), total nitrogen (TN), total phosphorus (TP), ammonia nitrogen (NH_4_^+^-N), nitrate nitrogen (NO_3_^–^N), soil available phosphorus (SAP), soil organic carbon (SOC), readily oxidizable carbon (ROC). The SBD and SWC were measured using the ring knife, respectively. Soil pH was determined using a pH meter (PHS-3C, INESA Inc., China). The concentrations of TC, TN, TP, NH_4_^+^-N and NO_3_^–^N were determined, using an elemental analyzer (Costech ECS 4024 CHNSO, Picarro, California, U.S.A.) and automatic chemical analyzer (Smartchem 4024, AMS Group, Italy), respectively. The concentrations of SAP, SOC and ROC contents were measured, using a continuous flow analyzer (AA3, Seal, Germany) and the potassium dichromate oxidation colorimetric method and potassium permanganate oxidation method, respectively (**Table S1**) (State Forestry Bureau, 1999; Guo et al., 2022).

### 2.5 Statistical analyses

The effect of sites on leaf stomatal and anatomical traits of moso bamboo were studied by one-way ANOVA. Mean comparisons were performed using the Duncan’s multiple range test at a probability level of 0.05. All values presented are means ± SD. Then, linear mixed-effect models were used to study the relationship among climate, leaf stomatal and anatomical traits of moso bamboo, and plot nested within site (random effects) (Bartholomew et al., 2022). Spearman correlation coefficients were calculated between soil environmental factors, stomatal and anatomical traits. The trait coordination network of moso bamboo was described by statistically significant correlations between stomatal and anatomical traits (Xie et al., 2022). Nodes in the network represent leaf stomatal and anatomical traits, and edges indicate correlations between traits. Only significant correlations are shown in the network (*P* < 0.05). Network centrality was characterized by degree (the number of edges connected to a node) and weighted degree (the sum of the edge weights of the adjacent edges of each node) (Messier et al., 2017). All variables were checked for normality and log-transformed when necessary. All statistical analyses were carried out using R-4.0.3 (R Core Team, 2020). The used R packages include ggplot2 v3.3.5 (Wickham, 2016), ggpubr v0.4.0 (Kassambara, 2020), ggsignif v0.6.3 (Ahlmann-Eltze and Patil, 2021), ggpmisc v0.4.5 (Aphalo, 2021), psych v2.1.9 (Revelle, 2017), rcompanion v2.4.15 (Mangiafico and Mangiafico, 2017), igraph v1.2.7 (Csardi, 2013), lme4 1.1-27.1 (Bates, 2010), lmerTest 3.1-3 (Kuznetsova et al., 2017).To explore the causal relationships among environment, leaf stomata and anatomical traits, and GPP, we used structural equation models (SEM) to estimate the path coefficients and variatiton of the dependent variables. We hypothesized that the environment affects leaf stomatal and anatomical traits, and ultimately affects GPP. A causal relationship between the variables was determined based on previous knowledge and preliminary qualitative studies. SEM was analyzed using Amos software ver. 23.0, and the model was evaluated using the maximum likelihood method (Arbuckle, 2014). The model parameters (0≤ χ2/df ≤3, RMSEA < 0.05, GFI > 0.9, CFI > 0.9, *P* > 0.05) were used to evaluate the fitting effect of the model (Lavorel and Grigulis, 2012; Ali et al., 2019).

## 3 Results

### 3.1 Site characteristics

Site significantly affected the soil physicochemical properties of moso bamboo (**Table S2**). For soil physical properties, CN areas had the highest SWC, XY and TJ areas had the lowest, while SBD showed the opposite trend. For soil chemical properties, the content of TC, TN, TP, NH_4_^+^-N, SOC, ROC and SAP were higher in the CN area, while the soil nutrient content was lower in the XY area (**Table S2**).

### 3.2 Leaf stomatal and anatomical traits

**Figure 3:**
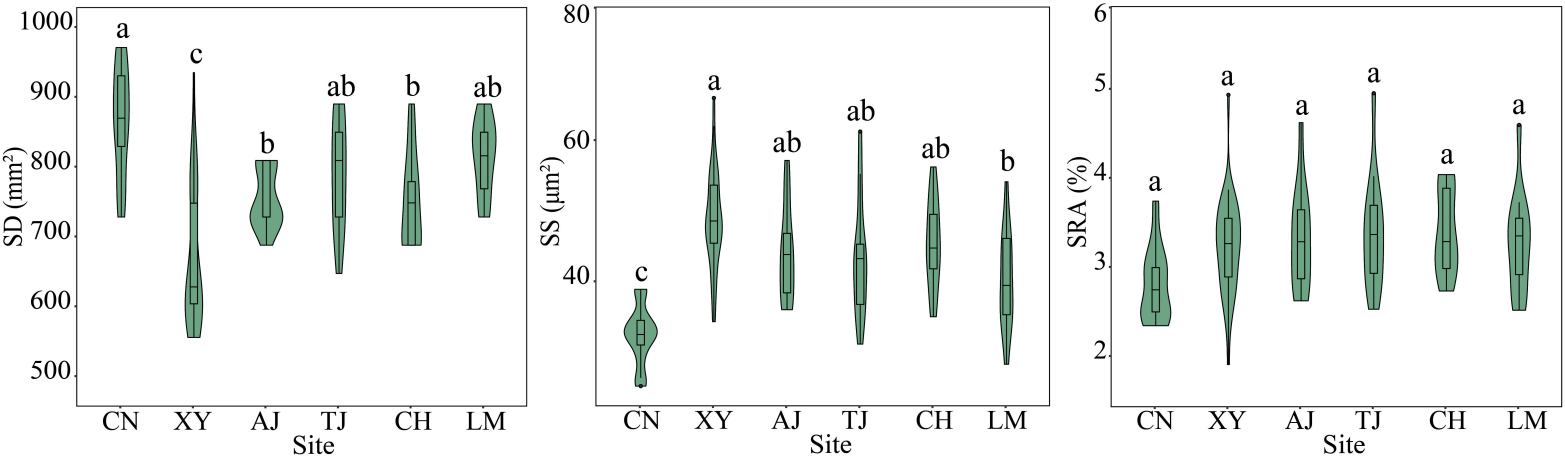
Leaf stomatal traits of moso bamboo forests in different sites. AJ, Anji; CH, Conghua; CN, Changning; LM, Longmen; TJ, Taojiang; XY, Xinyang. Different letters indicate significant differences among sites at the 0.05 level.

**Figure 4:**
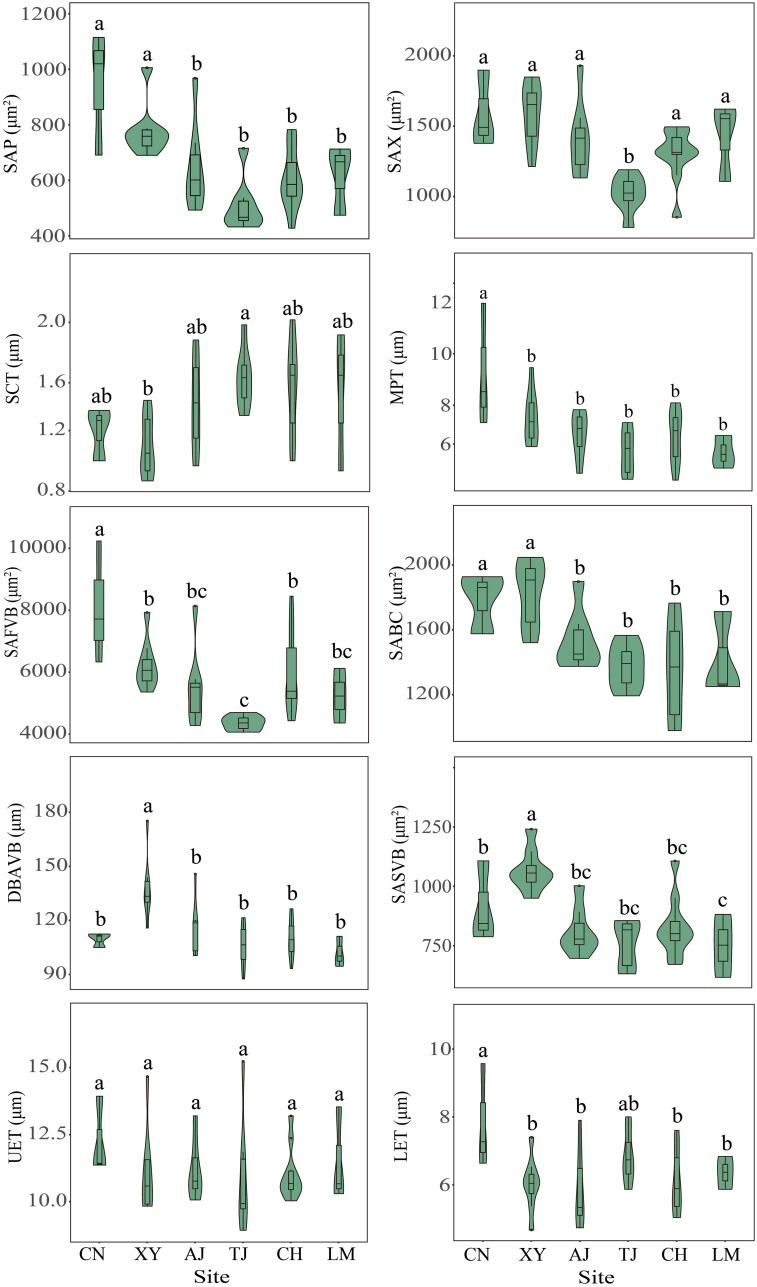
Leaf anatomical traits of moso bamboo forests in different sites. AJ, Anji; CH, Conghua; CN, Changning; LM, Longmen; TJ, Taojiang; XY, Xinyang. Different letters indicate significant differences among sites at the 0.05 level.

In general, site significantly affected the bamboo leaf stomatal and anatomical traits (*P* < 0.05) (**Figures 3**, **4**, **Table S3**). In terms of leaf stomatal traits, the northwestern site CN had significantly higher SD and lower SS than the other sites. In contrast, the northern site XY showed the opposite results. In terms of leaf anatomical traits, the northwest site CN had the highest SAP, MPT, SAFVB, and LET, and the northern site XY had the highest SAX, SABC, DBAVB, and SASVB, and both sites had lower SCT. While the central site TJ had the highest SCT and lowest SAX and SAFVB, the other sites had no significant differences in leaf stomatal and anatomical traits (*P* > 0.05) (**Figures 3**, **4**).

### 3.3 Trait coordination network

**Figure 5:**
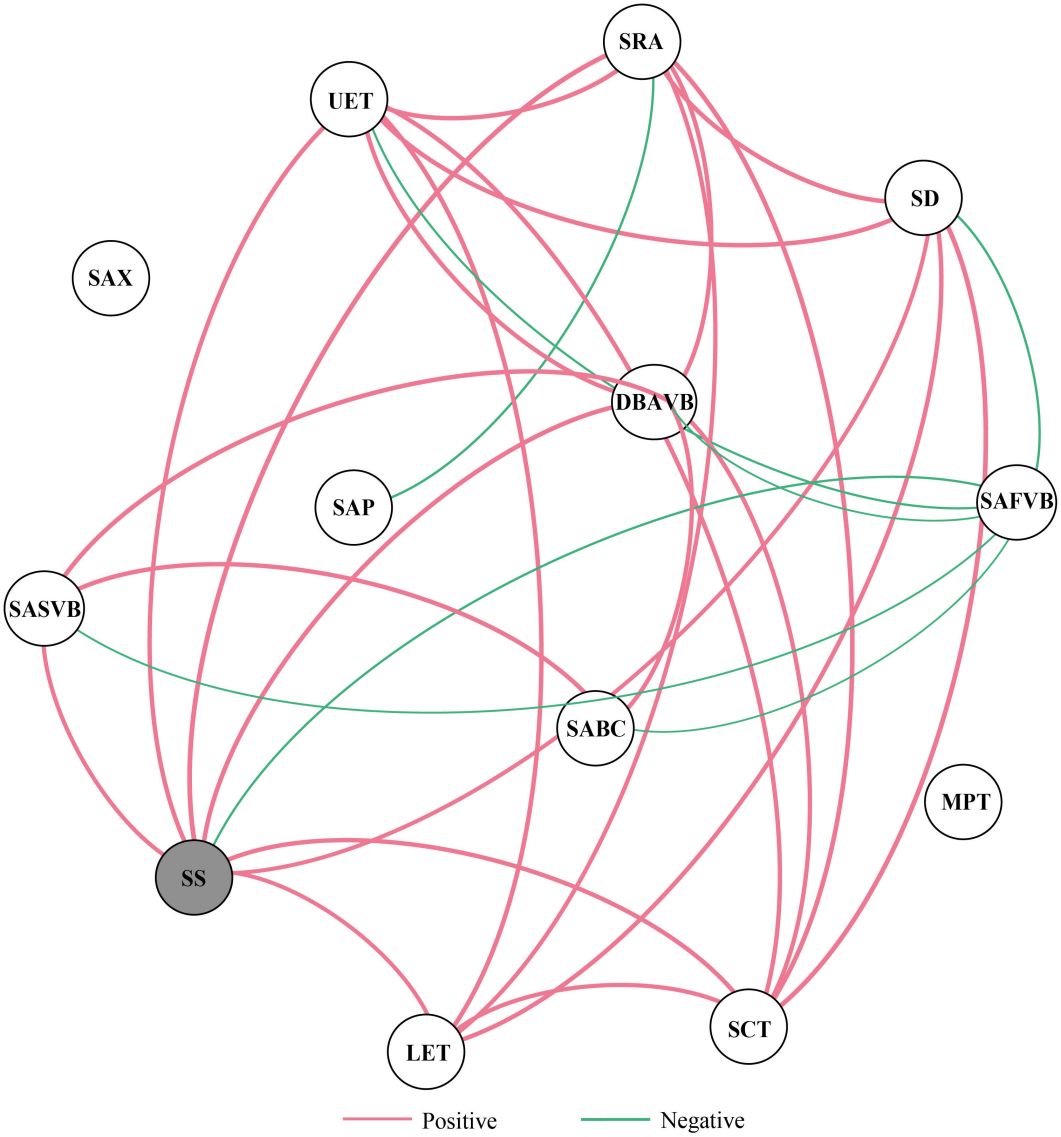
Trait correlation network of stomatal and anatomical traits of moso bamboo. Note: The red and green edges indicate positive and negative correlations, respectively. The thickness of the edges indicates the strength of the correlation. Traits marked by gray circles show the high centrality values in terms of degree and weighted degree.

At the regional scale, the average degree of the trait coordination network was 4.615 (13 nodes, 30 edges), and SS showed the highest centrality in the network (**Figure 5**, **Table S4**). The positive correlations were dominant in the trait coordination network, but all leaf stomatal and anatomical traits associated with SAFVB showed negative correlations. In addition, SAX and MPT were not significantly correlated with other leaf stomatal and anatomical traits (**Figure 5**).

### 3.4 Leaf stomatal and anatomical traits in relation to environmental conditions

Overall, leaf stomatal traits were limited by SR, and leaf anatomical traits were mainly affected by MAP and SR (**Table 2**). SR was significantly negatively correlated with SD, LET, and MPT, and significantly positively correlated with SS and SRA (*P* < 0.05). MAP was significantly negatively correlated with SAP, SASVB, MPT, and SABC, and significantly positively correlated with SCT (*P* < 0.05). Moreover, MAT was significantly negatively correlated with DBAVB of moso bamboo (*P* < 0.05).

**Table 2:** Relationship between climate and leaf traits (stomatal and anatomical traits) of moso bamboo analyzed using linear mixed-effects models.

Leaf traits		MAT		MAP		SR	
		R^2^	*P*	R^2^	*P*	R^2^	*P*
**Stomatal traits**	**SD**	0.12	0.29(+)	0.01	0.81(+)	0.24	***P*<0.05**(-)
	**SS**	0.06	0.49(-)	0.01	0.76(+)	0.38	***P*<0.05**(+)
	**SRA**	0.00	0.97(+)	0.10	0.13(+)	0.16	***P*<0.05**(+)
**Anatomical traits**	**SAFVB**	0.00	0.88(+)	0.12	0.31(-)	0.28	0.07(-)
	**SAP**	0.02	0.67(-)	0.31	***P*<0.05**(-)	0.20	0.15(-)
	**SAX**	0.01	0.73(-)	0.07	0.39(-)	0.01	0.72(-)
	**SASVB**	0.14	0.23(-)	0.29	***P*<0.05**(-)	0.00	0.87(-)
	**DBAVB**	0.30	***P*<0.05**(-)	0.23	0.10(-)	0.05	0.50(+)
	**UET**	0.00	0.78(+)	0.02	0.48(-)	0.10	0.12(-)
	**LET**	0.01	0.75(+)	0.06	0.37(-)	0.30	***P*<0.01**(-)
	**SCT**	0.06	0.36(+)	0.17	***P*<0.05**(+)	0.01	0.69(+)
	**MPT**	0.02	0.70(-)	0.26	***P*<0.05**(-)	0.24	***P*<0.05**(-)
	**SABC**	0.18	0.18(-)	0.49	***P*<0.01**(-)	0.05	0.55(-)

The part in bold indicates significant correlations, P < 0.05 significant difference, P < 0.01 highly significant difference. The direction (+ or -) of the correlation is indicated in parentheses. SD, stomatal density; SS, stomatal size; SRA, stomatal relative area; SAFVB, sectional area of first-vascular bundle; SAP, sectional area of phloem; SAX, sectional area of xylem; SASVB, sectional area of second-order vascular bundle; DBAVB, distance between adjacent vascular bundle; UET, upper epidermal thickness; LET, lower epidermal thickness; SCT, stratum corneum thickness; MPT, mastoid process thickness; SABC, sectional area of bulliform cell.

Values of R^2^ and *P*-level are given.

**Figure 6:**
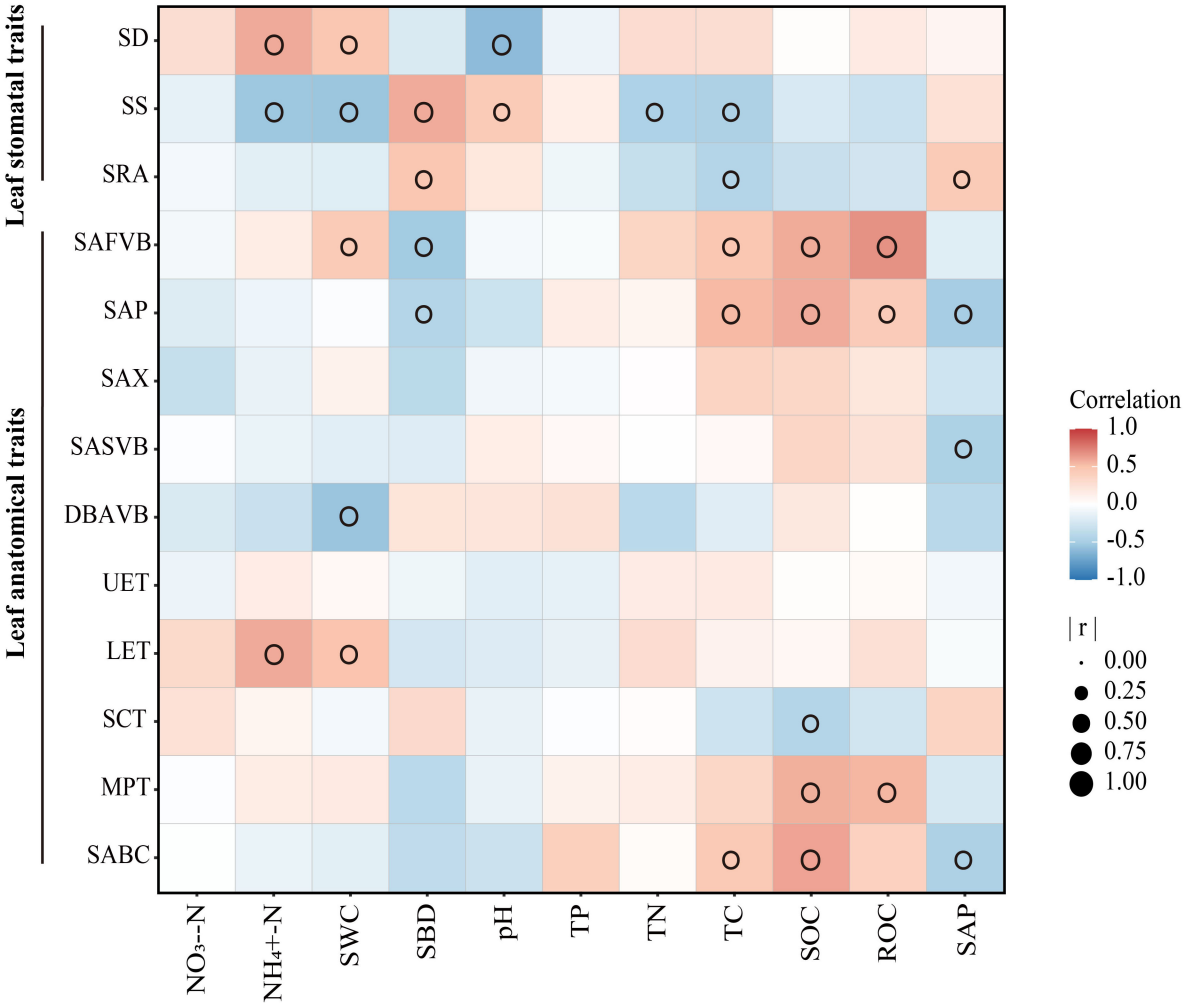
Relationship between soil and leaf traits of moso bamboo. Soil properties: SWC, soil water content; SBD, the soil bulk density; TC, total carbon; TN, total nitrogen; TP, total phosphorus; NO_3_^–^N, nitrate nitrogen; NH_4_^+^-N, soil ammonia nitrogen; SOC, soil organic carbon; ROC, readily oxidizable carbon; SAP, soil available phosphorus. Leaf traits: SD, stomatal density; SS, stomatal size; SRA, stomatal relative area; SAFVB, sectional area of first-vascular bundle; SAP, sectional area of phloem; SAX, sectional area of xylem; SASVB, sectional area of second-order vascular bundle; DBAVB, distance between adjacent vascular bundle; UET, upper epidermal thickness; LET, lower epidermal thickness; SCT, stratum corneum thickness; MPT, mastoid process thickness; SABC, sectional area of bulliform cell. Only significant correlations are shown in the figure (p < 0.05).

Generally, leaf stomatal and anatomical traits of moso bamboo were affected by soil factors (**Figure 6**). SWC, SBD and TC were the main factors that affected leaf stomatal and anatomical traits. The SS responded most strongly to the soil environment in leaf stomatal traits, while SAFVB and SAP were more sensitive to the soil environment in leaf anatomical traits.

### 3.5 The relationship among environmental factors, leaf stomatal and anatomical traits, and GPP

**Figure 7:**
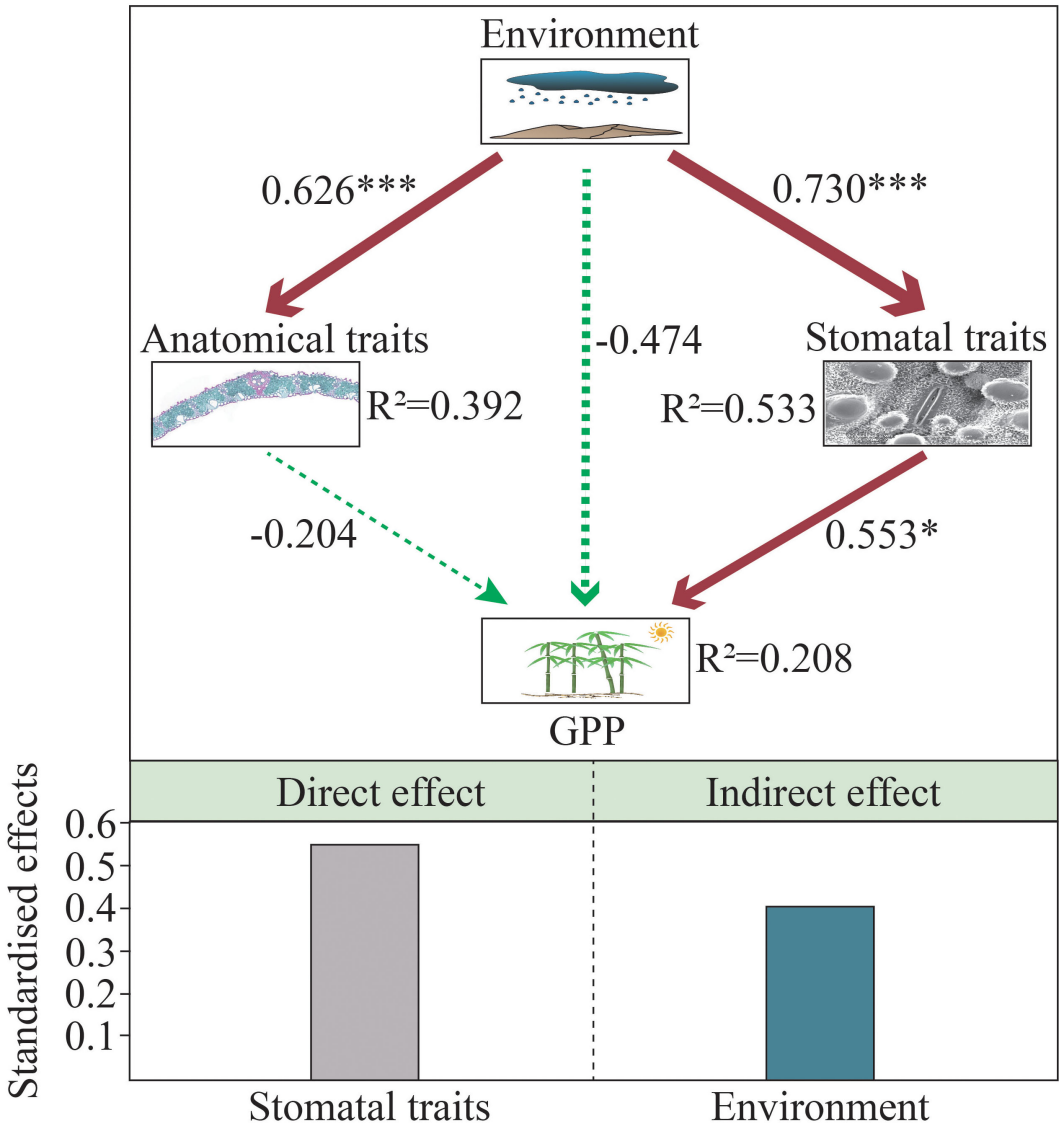
Structural equation modeling (SEM) showing the effects of environment, stomatal and anatomical traits on GPP. The red and green arrows represent the positive and negative relationships between the variables, respectively. The width of the arrow is proportional to the intensity, and the number next to the arrow represents the standardized path coefficient.

SEM analyses showed a significant positive correlation between environment and leaf traits stomata and anatomical traits (**Figure 7**). The effects of the environment on leaf stomata and anatomical traits were similar, and standardized path coefficients were 0.730 and 0.626, respectively. SEM revealed a direct positive effect of leaf stomatal traits on GPP (standardised direct effect = 0.553), while anatomical traits had no significant effect on GPP. Furthermore, the environment exerted indirect control on GPP through its positive effect on stomatal traits (standardized indirect effect = 0.404). There was no direct effect of environment on GPP, and leaf stomatal traits may have mediated the direct effect of environment on GPP. Overall, environment explained 53.3% and 39.2% of the variation in leaf stomatal and anatomical traits, respectively; leaf stomatal traits were the key driver explaining regional-scale variation in GPP, explaining 20.8% of GPP variation (**Figure 7**).

## 4 Discussion

### 4.1 Regional variations of stomatal traits and driving factors

Consistent with our second hypothesis, we found that the environment (climate and soil) significantly affected the leaf stomatal traits of moso bamboo (**Figures 6**, **7**, **Table 2**). SR was significantly negatively correlated with SD, and positively correlated with SS and SRA, indicating that bamboo could adapt to the external environment by regulating stomatal traits (**Table 2**). In other case, the SD of sedge was positively correlated with temperature and precipitation in the Eurasian Arctic, while the SS was the opposite (Stenström et al., 2002). The studies have shown that SD and SS were negatively correlated with precipitation and temperature, respectively, this difference may be related to genetic factors, leaf age, leaf vein density, altitude, etc. (Batos et al., 2010; Bertolino et al., 2019; Du et al., 2021; Amitrano et al., 2022). In this study, soil nutrients and moisture affected leaf stomatal traits of moso bamboo, and the SS of moso bamboo was more sensitive to soil variables (**Figure 6**). A previous study showed that SD and SRA were negatively correlated with soil nutrients (C, N and P) and pH, while SS showed the opposite trend (Liu et al., 2017). Sun et al. (2021) found that SS and SRA were significantly negatively correlated with C, N, and P, while SD showed the opposite trend. The effects of different environmental variables on leaf stomatal traits may be the result of their combined effects (Larcher et al., 2015; Du et al., 2021). SEM showed that leaf stomatal traits were the key drivers explaining the regional-scale variation of GPP (**Figure 7**). It was shown that stomatal density was positively correlated with ecosystem productivity, explaining 51% of its variation (Wang et al., 2015). To improve the prediction accuracy of the model, leaf stomatal traits should be included as new ecological parameters in the model for predicting ecosystem function.

### 4.2 Regional variations of anatomical traits and driving factors

Consistently as with the stomatal, traits, the environment (climate and soil) also significantly affected the leaf anatomical traits of moso bamboo (**Figures 6**, **7**, **Table 2**). General, MAT, MAP, and SR were significantly negatively correlated with leaf anatomical traits (**Table 2**). It was shown that leaf anatomical traits were significantly influenced by climate, especially MAT and MAP, which explained 33-72% of the large-scale total variation (He et al., 2017). Previous study have shown that grasses tend to increase vessel and vascular bundle diameters to resist drought stress (Wang et al., 2011; Wyka et al., 2019). This difference may be that the suitable zone of moso bamboo has not reached the level of drought stress, and its well-developed bamboo whip system has a strong water transport and storage function. In our study, the leaf anatomical traits of moso bamboo were affected by SWC, TC, SOC, ROC, SAP (**Figure 6**). It was found that leaf anatomical traits were influenced by soil moisture and nutrients in different habitats, and their total variation on leaf anatomical traits was about 20%, respectively, and leaf anatomical traits were more influenced by TN than TP (Wu et al., 2022). However, Lin et al. (2021) found that variation in leaf anatomical traits of *Cyclobalanopsis* was mainly controlled by soil pH. SEM showed that anatomical traits had no significant effect on GPP (**Figure 7**). In contrast, at the community scale, leaf anatomical traits of dicotyledonous plants were closely related to GPP (He et al., 2017). Therefore, the relationship between leaf anatomical traits of monocotyledons and ecosystem function may need more data to test this hypothesis.

### 4.3 Relationship between leaf stomatal and anatomical traits

We found that there may be a trade-off between leaf stomata and anatomical traits, and the traits were mainly synergistic (**Figure 5**). Qi et al. (2016) found that graminaceous plants adapt to environmental changes through the coordination between traits and have a strong overall adaptation to the environment. Previous studies have shown that high photosynthesis requires stomatal opening for gas exchange and transpiration for water loss, and plants may improve water transfer by regulating leaf anatomical traits to increase water use efficiency (Lawson and Blatt, 2014; Schneider et al., 2017). In accordance with our first hypothesis, leaf stomatal trait (SS) was the highest centrality value in moso bamboo, indicating that stomatal traits may play a dominant role in the rapid development of new bamboos (**Figure 5**). Previous studies have shown that SS shows the highest centrality value in humid area, while in regional scale, semi-humid and arid areas it is the stomatal opening ratio. Plants can adjust SD and SS in response to external environmental stresses, but when they reach a physiological threshold, the stomatal opening ratio may regulate changes in the trait coordination network along environmental gradients (Bertolino et al., 2019; Xie et al., 2022). Moreover, the leaf economic spectrum indicates growth condition-related trade-offs among leaf traits within a species, and thus leaf vein traits and other leaf traits should also be considered to better understand the acclimation and adaptation of plant photosynthetic mechanisms to environmental changes (Sack et al., 2013).

## 5 Conclusion

In line with our first hypothesis, there was a significant correlation between leaf stomatal traits and anatomical traits of moso bamboo. The leaf stomatal trait (SS) was the dominant trait adjusting the trait coordination network for environmental responses and adaptation, which may be a result of rapid growth rate and high photosynthetic efficiency of moso bamboos. Inconsistent with our second hypothesis, our study indicated that environmental factors (climatic and soil conditions) significantly influenced the spatial variation pattern of leaf stomatal and anatomical traits of moso bamboo. However, the key environmental factors affecting leaf stomatal differed with those for leaf anatomical traits. Moreover, leaf stomatal traits not anatomical traits significantly affected GPP variation at the regional scale. Our study provides direct evidence for the relationship between traits and bamboo ecosystem productivity. The present study contributes to better understanding the relationship among environment, leaf traits and ecosystem productivity of bamboo forests in a changing world.

## Data availability statement

The original contributions presented in the study are included in the article/**Supplementary Material**. Further inquiries can be directed to the corresponding authors.

## Author contributions

WG and LQ conceived the experimental design; WG and JZ contributed to the field and indoor experiments; WG, PC, and ML contributed to the data analysis and manuscript writing. All authors contributed to the article and approved the submitted version.
