# Supplementary material for: Leaf stomatal traits rather than anatomical traits regulate gross primary productivity of moso bamboo (Phyllostachys edulis) stands
Source: Front Plant Sci. 2023 Mar 14;14:1117564. doi: 10.3389/fpls.2023.1117564 (PMC10043342; doi:10.3389/fpls.2023.1117564)
Supplement: Supplementary file 1 [file Table_1.docx]

**Supplemental Tables**

**Table S1** Site information

| **Sampling**  **Sites** | **Longitude**  **(°E)** | **Latitude**  **(°N)** | **Altitude**  **(m)** | **MAT**  **(°C)** | **MAP**  **(mm)** | **SR**  **(kJ m^-2^ day^-1^)** | **PET**  **(mm year ^-1^)** | **AET(mm year ^-1^)** | **TS (standard deviation ×100)** | **PS** **(Coefficient of Variation)** | **GPP (kg C m^-2^ yr^-1^)** | **DBH**  **(cm)** | **Height**  **(m)** | **Soil**  **type** |
| --- | --- | --- | --- | --- | --- | --- | --- | --- | --- | --- | --- | --- | --- | --- |
| **CN** | 105°01'13" | 28°27'52" | 877.50 | 17.47 | 910.0 | 11973 | 88.00 | 92.50 | 708.40 | 70.89 | 1.18 | 9.20 | 13.06 | Yellow soil |
| **XY** | 114°04'33" | 31°49'14" | 490.00 | 14.83 | 942.5 | 13840 | 106.25 | 80.50 | 883.93 | 59.08 | 0.98 | 7.05 | 9.97 | Yellow-brown soil |
| **AJ** | 119°36'35" | 30°31'01" | 373.33 | 14.21 | 1144.4 | 14678 | 99.00 | 76.00 | 855.10 | 49.90 | 1.69 | 8.18 | 9.01 | Yellow-red soil |
| **TJ** | 112°04'41" | 28°19'38" | 252.50 | 16.50 | 1175.0 | 13560 | 99.00 | 87.75 | 843.04 | 46.48 | 1.15 | 10.72 | 14.22 | Red soil |
| **CH** | 113°48'13" | 23°44'09" | 292.50 | 20.00 | 1490.8 | 13515 | 103.50 | 80.50 | 599.04 | 66.76 | 1.39 | 8.05 | 12.65 | Red soil |
| **LM** | 113°50'56" | 23°38'06" | 552.50 | 20.20 | 1510.0 | 13472 | 99.25 | 88.25 | 586.45 | 66.69 | 1.53 | 9.27 | 14.41 | Yellow-red soil |

**Note:** **CN**: Changning; **XY**: Xinyang; **AJ**: Anji; **TJ**: Taojiang; **CH**: Conghua; **LM**: Longmen. **MAT**: mean annual temperature, **MAP**: mean annual precipitation, **SR**: solar radiation, **PET**: potential evapotranspiration, **AET**: actual evapotranspiration, **TS**: temperature seasonality, **PS**: precipitation seasonality, **GPP**: gross primary productivity, **DBH**: diameter at breast height.

**Table S2** Soil physicochemical properties of moso bamboo in different sites

| **Sites** | **SWC** | **SBD (g·cm^-3^)** | **pH** | **TC (g·kg^-1^)** | **TN (g·kg^-1^)** | **TP (g·kg^-1^)** | **NO_3_^—^N (mg·kg^-1^)** | **NH_4_^+^-N (mg·kg^-1^)** | **SOC (g·kg^-1^)** | **ROC (g·kg^-1^)** | **SAP (mg·kg^-1^)** |
| --- | --- | --- | --- | --- | --- | --- | --- | --- | --- | --- | --- |
| **CN** | **0.45±0.03a** | 0.82±0.03c | 4.12±0.02b | **65.05±3.07a** | **5.95±0.49a** | **0.05±0.00ab** | 9.25±0.40bc | **67.73±2.22a** | **42.28±4.05a** | **14.35±0.67a** | **3.41±0.19ab** |
| **XY** | 0.13±0.01c | **1.16±0.06a** | **4.88±0.18a** | 32.38±5.33b | 3.56±0.36b | **0.07±0.02a** | 8.33±0.47bc | 21.00±1.21d | **36.32±7.18ab** | 7.74±1.12bcd | 2.99±0.86b |
| **AJ** | **0.32±0.09ab** | 0.94±0.10bc | **4.79±0.12a** | 34.56±3.04b | **4.84±0.70ab** | **0.04±0.02ab** | 5.86±0.07c | 31.51±2.29c | **34.03±5.43abc** | 8.24±0.75bc | **5.11±1.21ab** |
| **TJ** | 0.24±0.01bc | **1.21±0.00a** | **4.64±0.10a** | 22.41±2.63c | **4.68±0.83ab** | **0.05±0.01ab** | **14.60±2.24a** | 41.77±3.73b | 20.54±3.18c | 6.21±1.17cd | **4.86±0.97ab** |
| **CH** | **0.37±0.02ab** | **1.13±0.03a** | **5.00±0.18a** | 27.63±0.72bc | **5.24±0.47ab** | 0.02±0.00b | 9.86±1.12b | 36.88±3.90bc | **28.55±2.27abc** | 10.01±0.62b | **5.28±0.67ab** |
| **LM** | **0.34±0.03ab** | **1.08±0.03ab** | **4.63±0.07a** | 32.94±1.34b | **5.11±0.30ab** | 0.03±0.01b | 7.68±0.96bc | 31.14±2.47c | 24.13±1.96bc | 5.43±0.29d | **5.70±0.46a** |

**Note:** **CN**: Changning; **XY**: Xinyang; **AJ**: Anji; **TJ**: Taojiang; **CH**: Conghua; **LM**: Longmen. In the table, **SWC** soil water content; **SBD** the soil bulk density; **TC** total carbon; **TN** total nitrogen; **TP** total phosphorus; **NO_3_^-^-N** nitrate nitrogen; **NH_4_^+^-N** soil ammonia nitrogen; **SOC** soil organic carbon; **ROC** readily oxidizable carbon; **SAP** soil available phosphorus; Different letters in the same column indicate significant differences at the 0.05 level.

**Table S3** Effect of site on stomatal and anatomical traits

| **Abbreviation** | **Traits** | **Site (S)** |
| --- | --- | --- |
| SD | Stomatal density | *** |
| SS | Stomatal size | *** |
| SRA | Stomatal relative area | ns |
| SAFVB | Sectional area of first-vascular bundle | ns |
| SAP | Sectional area of phloem | ** |
| SAX | Sectional area of xylem | * |
| SASVB | Sectional area of second-order vascular bundle | ns |
| DBAVB | Distance between adjacent vascular bundle | *** |
| UET | Upper epidermal thickness | ns |
| LET | Lower epidermal thickness | ns |
| SCT | Stratum corneum thickness | ** |
| MPT | Mastoid process thickness | ** |
| SABC | Sectional area of bulliform cell | ns |

**Note:** “*” significant difference (*P* < 0.05), “**” extremely significant difference (*P* < 0.01), “***” extremely significant difference (*P* < 0.001), “ns” no significant (*P* > 0.05).

**Table S4** Properties of the trait correlation networks in moso bamboo

| **Network properties** | **New bamboo** |
| --- | --- |
| **Number of nodes** | 13 |
| **Number of edges** | 30 |
| **Average degree** | 4.615 |
| **Average weighting degree** | 1.727 |
| **Graph density** | 0.385 |
| **Modularity** | 0.295 |
| **Average clustering coefficient** | 0.770 |
| **Average path length** | 1.527 |
